# Improving Sensor Adaptability and Functionality in Cartographer Simultaneous Localization and Mapping

**DOI:** 10.3390/s25061808

**Published:** 2025-03-14

**Authors:** Wonseok Jeong, Chanho Lee, Namyeong Lee, Seungwoo Hong, Donghyun Kang, Donghyeok An

**Affiliations:** 1 Department of Computer Engineering, Changwon National University, Changwon 51140, Republic of Korea; wsjung1218@gmail.com; 2 Department of Future Digital Solutions of Volvo Construction Equipment, Changwon 51706, Republic of Korea; chanho.lee@volvo.com (C.L.); namyeong.lee@volvo.com (N.L.); 3 Network Research Department, Electronics and Telecommunications Research Institute (ETRI), Daejeon 34129, Republic of Korea; swhong@etri.re.kr; 4 Department of Computer Engineering, College of IT Convergence, Gachon University, Seongnamsi 13120, Republic of Korea

**Keywords:** Cartographer, simultaneous localization and mapping, Robot Operating System, multi-node, real time

## Abstract

This paper aims to address sensor-related challenges in simultaneous localization and mapping (SLAM) systems, specifically within the open-source Google Cartographer project, which implements graph-based SLAM. The primary problem tackled is the adaptability and functionality of SLAM systems in diverse robotic applications. To solve this, we developed a novel SLAM framework that integrates five additional functionalities into the existing Google Cartographer and Robot Operating System (ROS). These innovations include an inertial data generation system and a sensor data preprocessing system to mitigate issues arising from various sensor configurations. Additionally, the framework enhances system utility through real-time 3D topographic mapping, multi-node SLAM capabilities, and elliptical sensor data filtering. The average execution times for sensor data preprocessing and virtual inertial data generation are 0.55 s and 0.15 milliseconds, indicating a low computational overhead. Elliptical filtering has nearly the same execution speed as the existing filtering scheme.

## 1. Introduction

Since the advent of Industry 4.0, intelligent and automated manufacturing technologies have rapidly evolved [1]. The requirements for robots used at diverse industrial sites have become increasingly sophisticated. In addition to stationary robots used in manufacturing lines, mobile robots such as vacuum cleaners and service robots are becoming more popular. Mobile robots can be utilized in high-risk environments, such as construction and disaster sites; however, autonomous map generation is required for autonomous movement. An interactive visual navigation (IVN) system based on reinforcement learning and task-related latent variable prediction has been proposed [2]. IVN employs a framework that learns from the agent’s actions and interactions with the environment, but it does not enable map construction. Maps can be constructed using simultaneous localization and mapping (SLAM), a map construction technique that records the distance traveled and predicts the robot’s own position without prior knowledge [3,4,5,6,7].

SLAM has versions that utilize various sensors such as light detection and ranging (LiDAR) sensors, cameras, and inertial measurement unit (IMU) sensors, and these versions are also differentiated by various algorithms and operating environments [8,9,10]. SLAM is primarily classified as either visual SLAM using cameras or LiDAR SLAM using LiDAR sensors. LiDAR SLAM is widely used because visual SLAM lacks a distance recognition capability and has low accuracy. Offline SLAM and online SLAM are distinguished based on whether the sensor data are collected in real time. Offline SLAM collects all sensor data before constructing a map, whereas online SLAM uses only sensor data received in real time to construct a map. Graph-based and filter-based SLAM are used to estimate the current position and map, which are SLAM’s main problems. Recently, graph-based SLAM has become a dominant approach. It addresses the primary location problem by representing the relevant information as nodes and constructing a map from the edges. Graph-based SLAM can incorporate various sensors, including LiDAR and IMU sensors, in the graph configuration, resulting in good sensor scalability and effective error minimization through the graph structure [11]. Popular open-source libraries that use graph-based SLAM include RTAB-Map, Cartographer, ORB-SLAM, and Hector SLAM [9,12,13]. Because we use LiDAR data for SLAM and Hector SLAM does not enable 3D mapping, we decided to use Google Cartographer, which is laser-based, rather than RTAB-Map and ORB-SLAM, which use a vision-based algorithm [14,15].

Google Cartographer (Cartographer) is an open-source library that uses graph-based SLAM. It uses branch-and-bound optimization techniques to reduce the amount of computation required. Consequently, for 2D SLAM, Cartographer can compute high-resolution maps of up to 5 cm in real time and integrates them with the Robot Operating System (ROS) environment. The ROS is an open-source framework for robotic applications [16]. Several functions and libraries are available, including hardware abstraction, message passing between components, and sensor data processing [17].

However, numerous issues arise when Cartographer uses bag files containing sensor data. First, Cartographer does not operate properly when the time between several sensor data points is not synchronized or when some sensor data fields are omitted. Second, when constructing a 3D map in real time, only the most recently measured 3D sensor data are displayed, rather than all sensor data measured to date. Third, utilizing multiple robots to create a map has advantages in terms of scalability and time efficiency. However, because Cartographer performs SLAM on a single node, numerous nodes cannot construct a single map. Finally, when filtering the sensor data, numerous data points are filtered based on their distances from the origin.

Existing experiments have been conducted to improve the SLAM performance using Cartographer [18,19,20,21,22,23,24,25,26]. Despite improvements to the Cartographer algorithm [18,27,28], it cannot process sensor timestamp synchronization or omitted sensor data. Cartographer has improved 3D mapping by enhancing the point cloud consistency [29]. However, improvements to the processing performance for the recorded sensor data and functional improvements for integration with ROS visualization (rviz) are required. Map construction strategies and path-planning algorithms based on multi-node SLAM have been proposed [30,31]. However, implementing multi-node SLAM in Cartographer has not yet been discussed.

This paper presents several schemes for improving Cartographer’s sensor data adaptability and functionality in an ROS2 environment. The main contributions of this study are as follows:

- We propose a time synchronization scheme for asynchronous sensor data.
- We propose a scheme for generating inertial data in order to address IMU data loss.
- We propose a scheme for expressing all measured sensor data in rviz while constructing a 3D map.
- We propose a scheme for enabling multi-node SLAM.
- we propose elliptical filtering, which filters data using the elliptic equation, to avoid over-filtering.

The remainder of this paper is organized as follows. Section 2 provides background information and outlines the structures of existing SLAM systems. Section 3 examines the limitations inherent in current SLAM systems. Section 4 delineates the architecture and functional implementation of the proposed SLAM system. Section 5 evaluates the proposed system, and Section 6 concludes the paper by discussing potential avenues for future research.

## 2. Background and Related Works

There are several popular open-source SLAM implementations, including RTAB-Map, ORB-SLAM, Hector SLAM, and Cartographer [9,13,14,15]. ORB-SLAM is a visual SLAM system that utilizes camera inputs [9]. It uses ORB characteristics to track and map the environment. However, unlike ORB-SLAM, our study was based on LiDAR SLAM. Hector SLAM is designed for quick mapping in indoor environments and uses LiDAR for 2D mapping. However, it does not support 3D mapping [13]. RTAB-Map and Cartographer enable both 2D and 3D mapping [14,15]. RTAB-Map is a visual SLAM system that uses camera inputs by default but can also use LiDAR as an option. Cartographer, on the other hand, is a LiDAR SLAM implementation that takes inputs from LiDAR. Table 1 compares the features of the existing SLAM implementations with the proposed approach. The SLAM implementations do not support multiple nodes, but the proposed approach does.

**Figure 1:**
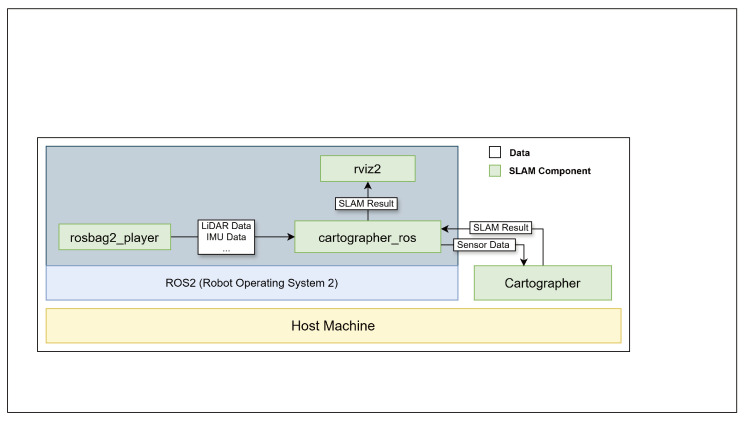
SLAM architecture and operation. Rosbag2_player sends LiDAR and IMU sensor data from the bag file to cartographer_ros. Cartographer performs SLAM using sensor data obtained from cartographer_ros and returns the results to cartographer_ros. The SLAM results are transferred from cartographer_ros to rvis2 and visualized.

**Figure 2:**
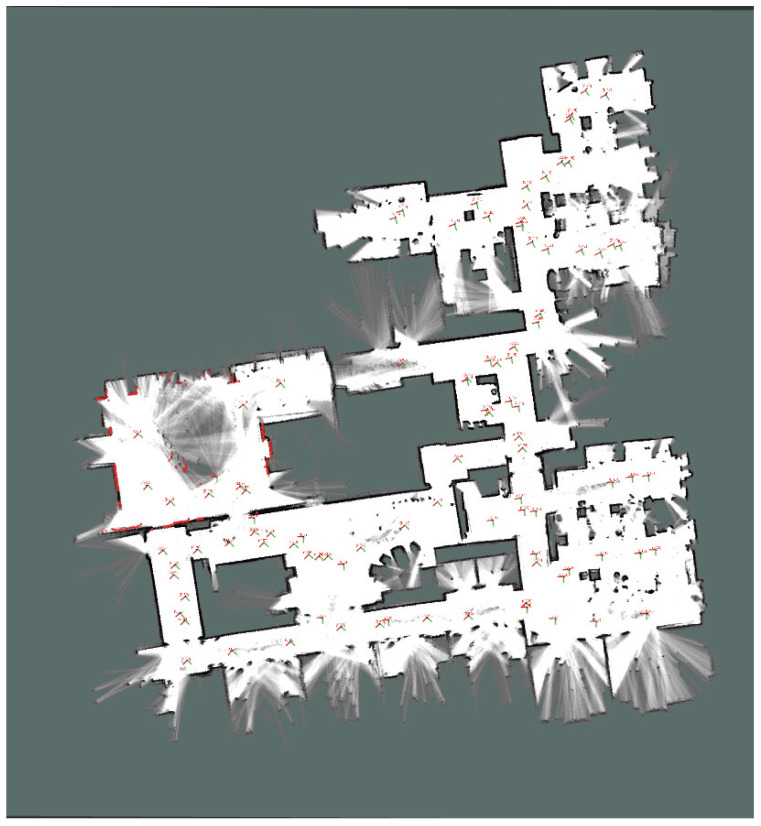
rviz2 visualization example.

Cartographer is a SLAM system developed by Google [14]. Figure 1 depicts the architecture and operation of a Cartographer-based SLAM system. Cartographer can obtain sensor data from a bag file containing a variety of sensor data, including those from LiDAR and IMU sensors. Because components in ROS2 can communicate using the publish–subscribe mechanism, rosbag2_player sends sensor data stored in the bag file to cartographer_ros. cartographer_ros includes cartographer_node, cartographer_occupancy_grid_node, and submaps_display. cartographer_ros transmits the sensor data to Cartographer, which executes graph-based SLAM and sends the results to cartographer_ros, which then sends the sensor data and SLAM results to rviz2 [32]. The Cartographer-based SLAM system then visualizes the map created using rviz2, as illustrated in Figure 2.

In [27], because the Cartographer SLAM algorithm, which performs loopback scan-to-map detection, exhibits errors in environments with few distinguishable characteristics, submap matching was used to address the errors. Preliminary matching and lazy decisions were utilized to improve the real-time performance. In [18], a multi-stage distance scheduler was proposed to increase Cartographer’s SLAM processing performance. The proposed scheme improved the local SLAM by adjusting the distance between the LiDAR sensor and the scan matcher’s search window. In [28], KP-Cartographer was proposed, a lightweight SLAM scheme for mapping and estimating locations using LiDAR data. Laser point cloud feature extraction and personal localization algorithms have been used in low-power mobile devices. However, previous studies were unable to handle scenarios in which the sensor timestamps disagreed or there were no inertial data.

In [29], an algorithm for continuous-time SLAM was proposed to improve Cartographer’s SLAM 3D mapping. The performance was improved by enhancing the global point consistency. However, greater processing performance for the recorded bag files and a scheme for connecting to rviz are required. A strategy for effective map construction using multi-robot systems in a communication-limited environment was proposed in [30]. Owing to limited communication resources, data transmission for grid map construction causes bottlenecks. The creation of a topology map for each robot reduces the amount of data transmitted. A system for creating and updating maps and path planning for a heterogeneous group of robots was proposed in [31]. Its client–server architecture improves the map accuracy. However, while existing research has proposed a scheme for multi-robot-based map construction, methods for multi-robot SLAM in Cartographer have not been proposed.

## 3. Sensor Adaptability Improvement

This section discusses the approaches for increasing sensor data flexibility. First, we analyze cartographic procedures in which timestamps are asynchronous or inertial data are missing. We then discuss approaches for timestamp synchronization and generating inertial data.

### 3.1. Analysis of Cartographer for Sensor Adaptability Improvement

In this section, we discuss asynchronous sensor data and the absence of inertial data.

#### 3.1.1. Asynchronization of Sensor Data

Cartographer can utilize various types of sensor data such as LiDAR and IMU data when performing graph-based SLAM. Although SLAM’s accuracy can be enhanced by integrating multiple sensors, the sensor data must be synchronized. Although Cartographer includes a synchronization process within its sensor data processing pipeline, the analysis revealed limitations in cases where synchronization requires substantial data modification.

Cartographer’s synchronization is based on the sensor data timestamps. In general, the sensor sets the timestamp of the data to the Unix time when it was logged. Algorithm 1 is pseudocode that depicts Cartographer’s method for processing sensor data. When two or more sensors sense at the same moment, but the times reported to Cartographer differ owing to network delays, the sensor data are synchronized using the sensor data’s timestamp and are processed chronologically. First, based on the sensor information, a queue for each currently active sensor is initialized. Sensor data S or the input sensor data are inserted into the corresponding queue. Then, synchronization starts if all sensor data queues contain more than one item of sensor data. For example, if there are two LiDAR sensor queues and one IMU sensor queue, but only one LiDAR sensor is operational, data are only fed into the associated LiDAR queue, and the synchronization operation is not performed. Sensor data with a timestamp that is later than that of the most recently input data from each sensor are used for synchronization. Sensor data with a timestamp that is older than the most recently input data from each sensor are filtered out. In Algorithm 1, the CommonStartTime variable stores the timestamp for the most recently input data from each sensor. The cur_data variable refers to the oldest data entered into all the sensor queues.
**Algorithm 1** Cartographer sensor data processing.1:**Init:** Queues.Initialize(∀ sensor)2:**Input:** sensor data *S*3: 4:CommonStartTime←−15:AddQueueData(*S*)6:**if** ∀Queues not empty **then**7:    **if** CommonStartTime=−1 **then**8:        CommonStartTime←max(∀Queues.Peek())9:    **end if**10:    **repeat**11:        cur_data←min(∀Queues.Peek())12:        cur_queue←GetQueue(cur_data)13:        **if** cur_data.timestamp > CommonStartTime **then**14:           AddSensorData(cur_data)15:        **end if**16:        cur_queue.pop()17:    **until** ∀Queues is empty18:**end if**19: 20:**Output:** All sensor data in Queues are synchronized in time order.

When using cartographic sensor data processing, issues may arise if the timestamps of the sensors differ in the execution environment. For instance, if a LiDAR sensor and an IMU sensor operate simultaneously but their timestamps differ by 300 s, SLAM will not execute for the first 5 min. Cartographer determines that the timestamps of LiDAR data and IMU data with 5 min intervals are the same and operates accordingly. The manual adjustment of the timestamps at the hardware level may not be feasible for solving the asynchronous timestamp problem.

#### 3.1.2. Absence of Inertial Data

When executing 3D SLAM, Cartographer is designed to require inertial measurement unit (IMU) data. The analysis results revealed that, when executing 3D SLAM, the IMU data queue is always initialized, as shown in Algorithm 1, and the IMU data are utilized for position estimation calculations. This approach is necessary to define the z-axis based on the gravity measured by the IMU sensor and to derive the roll and pitch values for accurate position estimation. However, if 3D SLAM is applied to a robot that moves at a steady pace, measurements from some sensors are superfluous. Furthermore, if some inertial sensors do not function properly, some of the required IMU data may not be measured. For example, if the gravity sensor fails due to an impact when the robot is in motion, SLAM will not work because there are no IMU sensor data. However, Cartographer does not offer the option to disable IMU data when performing 3D SLAM. Thus, a solution for sensor adaptability is required.

### 3.2. Proposed Scheme for Sensor Adaptability Improvement

**Figure 3:**
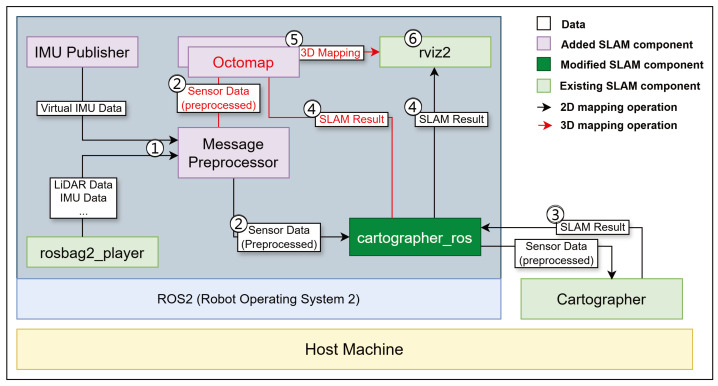
Proposed SLAM architecture. 1. LiDAR and IMU sensor data are delivered from the bag file to message preprocessor. Simultaneously, virtual inertial data are generated if any inertial data are missing. 2. Synchronized sensor data are sent to cartographer_ros and Octomap. 3. Cartographer performs SLAM, and the results are sent to cartographer_ros. 4. The SLAM results are delivered from cartographer_ros to rviz2 and Octomap. 5. When 3D mapping is used, Octomap sends 3D mapping results to rviz2. 6. The rviz2 visualizes the results.

In this section, we discuss sensor data time synchronization and virtual inertial data generation. Figure 3 shows the architecture of the proposed SLAM system. Rosbag2_player delivers LiDAR and IMU sensor data from the bag file to cartographer_ros. Simultaneously, the IMU Publisher generates virtual inertial data if any inertial data are missing. Then, the message preprocessor performs the time synchronization of the sensor data.

#### 3.2.1. Sensor Data Time Synchronization

**Figure 4:**
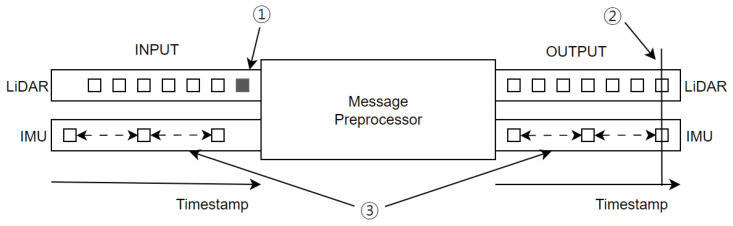
Sensor data preprocessing. 1. If the LiDAR sensor is a reference sensor, the reference timestamp is set to the first input sensor data point of the LiDAR data. 2. The timestamp value of the IMU data’s first sensor data point is assigned as the reference timestamp. 3. The timestamp is then reset using the sampling rate of the IMU sensor data.

To improve the synchronicity of timestamps between sensor data, we propose a message preprocessor node. The message preprocessor node is added to synchronize various sensor data before they are sent to cartographer_ros. Figure 4 and Algorithm 2 show the message processor’s synchronization process. We assumed that the initial data from each sensor are sensed at the same time point. The message preprocessor takes the original sensor data values as the input and outputs the synchronized sensor data values. First, the message preprocessor arbitrarily designates a sensor as the reference for timestamp synchronization. A LiDAR sensor, for example, can be used as the reference sensor. If the type of input sensor data comes from a reference sensor and the reference timestamp has not yet been established, the timestamp of the associated sensor data is used as the reference timestamp. For example, if the LiDAR sensor is a reference sensor, the reference timestamp is set to the first input sensor data point of the LiDAR data, as shown in Step 1 of Figure 4. If the input sensor data do not come from a reference sensor and the reference timestamp has already been set, the sensor data’s timestamp is set to the reference timestamp plus the measurement time from the reference timestamp setting to the current time. For example, as shown in Step 2 of Figure 4, the timestamp value of the IMU data’s first sensor data point is assigned as the reference timestamp. As Step 3, the timestamp is then reset using the sampling rate of the IMU sensor data. Then, the synchronized data are passed to cartographer_ros for normal SLAM operation.

#### 3.2.2. Virtual Inertial Data Generation

**Figure 5:**
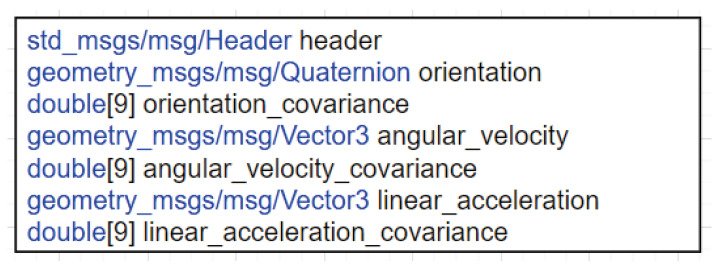
Imu.msg format.

To address the absence of inertial data, we propose a new ROS2 node termed the IMU Publisher, which generates virtual inertial data, as illustrated in Figure 3. The format of the IMU data follows that of the ROS2 sensor_msgs/msg/Imu.msg format. Figure 5 illustrates the composition of the Imu.msg format [33]. The header field contains the timestamp value, which indicates the time at which the data were generated, and the frame_id value, which represents the associated coordinate frame. The orientation, angular_velocity, and linear_acceleration fields, respectively, represent the direction, angular velocity, and linear acceleration components as measured using the IMU sensor. The covariance values corresponding to each component are located in the orientation_covariance, angular_velocity_covariance, and linear_acceleration_covariance fields.
**Algorithm 2** Sensor data timestamp synchronization.1:**Input:** sensor data *S*2:**Initialize:**3:   reference_sensor←oneofthesensors4:   reference_timestamp←underfind5: 6:**repeat**7:    **if** S.type=reference_sensor **then**8:        **if** reference_timestamp=underfind **then**9:           reference_timestamp←S.timestamp10:           timer.start()11:        **end if**12:    **else**13:        **if** reference_timestamp≠underfind **then**14:           S.timestamp←reference_timestamp+ timer.current_time()15:        **end if**16:    **end if**17:**until** termination

**Figure 6:**
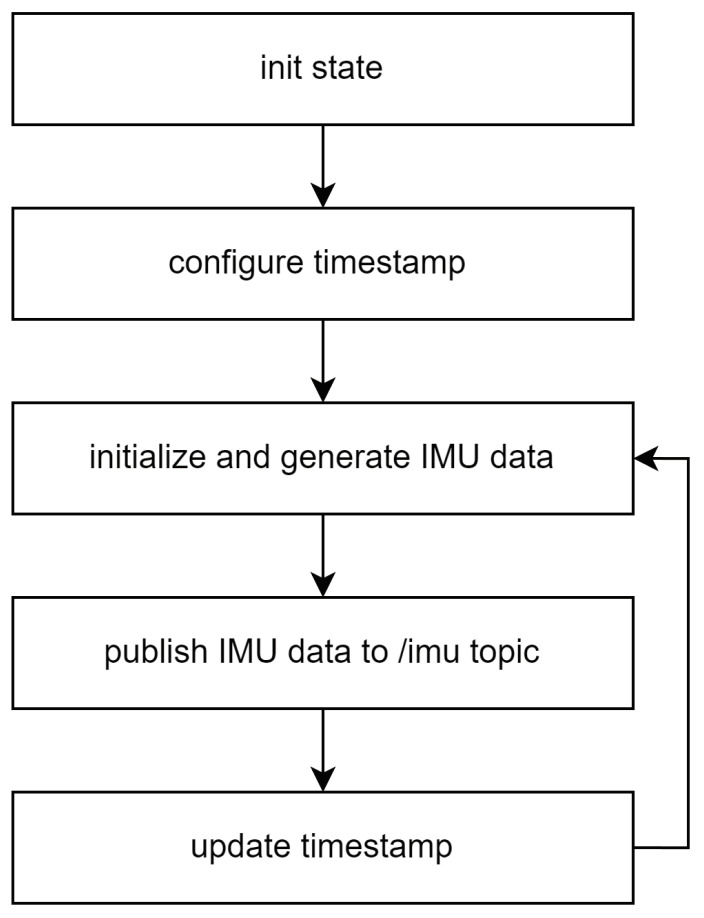
Flowchart of IMU publisher.

Figure 6 illustrates the flowchart of the IMU Publisher. The IMU Publisher starts operating when the user enters a timestamp. The timestamp value of the generated IMU data is set as the input timestamp value, allowing the user to generate a message at the desired time. Then, only the z component of linear_acceleration is set to a gravitational acceleration of 9.8 m/s^2^ while the other components are set to their default values, assuming that some IMU data may have been lost due to the inertial sensor failure. The generated IMU data are published periodically to a designated topic (/imu) within the ROS system. The virtual inertial data are passed to the message preprocessor for timestamp synchronization.

## 4. Functionality Improvement

This section discusses the approaches for functionality improvement. First, we analyze limitations for real-time 3D mapping and map accuracy. We then discuss approaches for real-time 3D mapping, multi-node SLAM, and elliptical filtering.

### 4.1. Cartographer Analyzed for Functionality Improvement

In this section, we discuss how to disable real-time 3D mapping, support single-node SLAM, and use distance-based filtering.

#### 4.1.1. Limitation for Real-Time 3D Mapping

**Figure 7:**
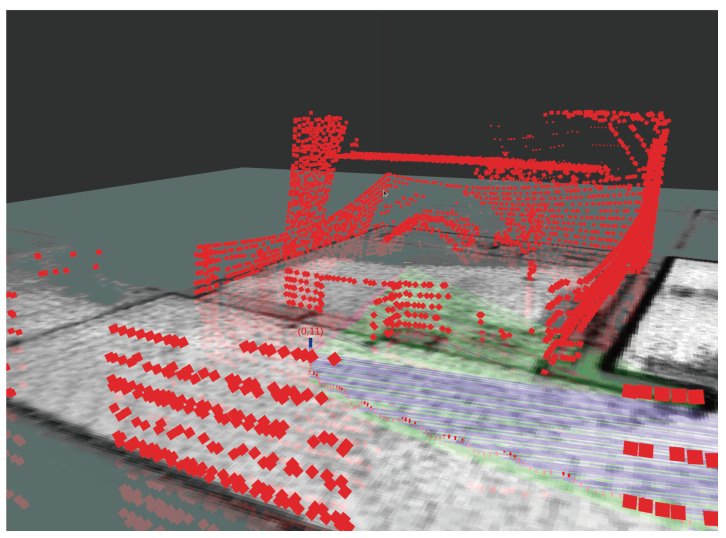
3D SLAM result.

The 3D terrain map generated in real time by Cartographer does not continuously record 3D sensor data. It represents only the 3D sensor data at the current time, as shown in Figure 7. A scheme for extracting all sensor data into a single 3D terrain map was proposed for Cartographer [34]; however, it has two constraints.

First, Cartographer does not provide real-time functionality to construct 3D terrain maps. A 3D terrain map can be constructed using Cartographer’s asset writer node, which requires bag and PBstream files. The pbstream file contains the results and status data processed by Cartographer’s SLAM operation [34]. Using both bag and pbstream files can result in a more accurate and high-resolution output. However, because the pbstream file is generated by the SLAM operation based on the bag file, additional time is required. Therefore, constructing a 3D terrain map in a real-time sensor environment is challenging.

Second, Cartographer generates a 3D terrain map in either the PCD or PLY file format [34]; however, these files cannot be directly visualized using rviz, the Cartographer SLAM system’s visualization tool. To visualize the generated map, a different viewer program that supports the corresponding file format is required, or Cartographer should be modified to publish the file as a PointCloud2 topic, allowing for rviz visualization.

#### 4.1.2. Limitation for Map Accuracy

Several nodes must be used to improve the accuracy and efficiency of map construction. In Cartographer, trajectory_builder generates a map using a trajectory that includes the estimated robot position and other sensor data. However, since SLAM is performed using a single robot, only one trajectory is typically defined for each map. Therefore, Cartographer supports single-node SLAM, and it is not possible to support multi-node SLAM.

Distance measurement sensors such as LiDAR and laser sensors require data filtering to provide accurate SLAM results based on their positions. For instance, if a portion of the robot’s body is recorded by the sensor, the SLAM results generated from the data will always indicate objects near the robot’s location, unlike in a real environment. To resolve this issue, Cartographer filters the sensor data [35]. However, because the filtering range is determined by the radius of a circle originating from a central point, the further the filtering target point is from the origin, the more the sensor data loss increases as the distance from this origin increases.

### 4.2. Proposed Scheme for Functionality Improvement

This section discusses real-time 3D mapping, multi-node SLAM, and elliptical filtering. In Figure 3, the message preprocessor transmits time-synchronized sensor data to cartographer_ros. Cartographer_ros performs data filtering and delivers the results to Cartographer. Cartographer performs SLAM, and the results are sent to Rviz2 via cartographer_ros. When 3D mapping is used, the message preprocessor sends sensor data to Octomap, whereas cartographer_ros sends SLAM results to Octomap. Octomap sends 3D mapping results to rviz2, which visualizes the results.

#### 4.2.1. Real-Time 3D Terrain Mapping

We propose a scheme to integrate the OctoMap library with Cartographer to enable real-time 3D terrain mapping, as shown in Figure 3. OctoMap is an open-source library implemented using a 3D occupancy grid mapping approach [36]. OctoMap can adjust the resolution, optimize memory usage, and perform 3D mapping using 3D PointCloud data from LiDAR sensors. Furthermore, it visualizes a generated 3D map using rviz. The preprocessed sensor data from the message preprocessor are then sent to cartographer_ros and OctoMap. Cartographer_ros transmits the SLAM results to OctoMap and rviz2. Then, the integration of OctoMap and Cartographer proceeds by supplying 3D PointCloud data to OctoMap and synchronizing the TF (Transform) information from Cartographer with OctoMap.

An important task in integration is the synchronization of TF information. TF information refers to the transformation relationships between coordinate frames in the ROS [37]. Cartographer calculates the robot’s position at a specific point in time and generates TF information. The generated TF information is distinguished by a coordinate system and a timestamp and stored in tf2_buffer. Because tf2_buffer stores and manages TF information, it can share it with ROS nodes. At this time, the synchronization of the timestamps of the two TF information is required for the synchronization of Cartographer and OctoMap.

When executing SLAM using bag files in an offline sensor environment, the timestamp information used by the two libraries may be inconsistent. OctoMap requests TF information based on the 3D sensor data’s timestamps. However, Cartographer sets the timestamp of the TF information to the most recent time generated from either the current node’s time or the time when Cartographer’s extrapolator performed the last prediction. In general, the current node’s time is chosen first because choosing the most recent time is more efficient because of Cartographer’s interpolation. However, in sensor-offline conditions, an exception may arise if the time zone of Cartographer’s node is set later than the sensor data’s timestamp.

**Figure 8:**
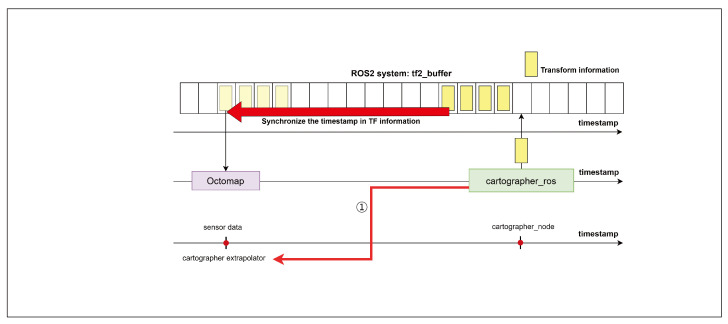
Exceptional case in sensor-offline environment.

Figure 8 illustrates the generation and requesting of TF information in such an exceptional case based on timestamps. Because the timestamp of all the generated TF information corresponds to the Cartographer node’s time, OctoMap cannot receive the appropriate TF information. In this case, as shown in Step 1 of Figure 8, the issue can be resolved by modifying Cartographer to set the timestamp of the generated TF information to the last execution time of the extrapolator. The extrapolator’s final execution time can be identified as being in the same time zone as the timestamp of the 3D sensor data, thereby resolving the issue of synchronizing the TF information with OctoMap.

#### 4.2.2. Multi-Node SLAM

We improved the code for a multi-node SLAM system to allow trajectory_builder to construct a single integrated map from numerous trajectories. Algorithm 3 depicts the multi-node SLAM processing. The start_trajectory and finish_trajectory functions in Cartographer were used to create and terminate the trajectories, respectively [38]. The start_trajectory function utilizes configuration_directory, configuration_basename, use_initial_pose, initial_pose, and relative_to_trajectory_id as an argument. Then, it generates a new trajectory and returns the generated trajectory ID. The trajectory’s initial position can be set using initial_pose and relative_to_trajectory_id, depending on the value of use_initial_pose. To terminate the trajectory, the finish_trajectory function uses the trajectory ID as the input and returns the status value. In the algorithm, TrajectoryID represents the current robot’s trajectory ID. The following actions are repeated sequentially from the first robot to the Nth robot. The *i*th trajectory, trajectory *_i_*, is generated by using the start_trajectory function with configuration *_i_*, and the generated trajectory ID is saved in TrajectoryID. The robot’s trajectory information is then displayed on the map. When the robot’s operation is complete, TrajectoryID is used as an argument for the finish_trajectory function, which terminates the current trajectory. The i+1th trajectory is then used to construct a map. The previously generated map is preserved. This allows sensor data from several robots to be combined into a single integrated SLAM system.
**Algorithm 3** Multi-Node SLAM Processing1:N = 1, 2, 3, …2:*i* = 13:Robot*_N_*: N-th operation robot4:Configuration*_N_*: configuration_directory*_Robot_N__*5:   configuration_directory*_Robot_N__*6:   configuration_basename*_Robot_N__*7:   use_initial_pose*_Robot_N__*8:   initial_pose*_Robot_N__*9:   relative_to_trajectory_id*_Robot_N__*10:**for**i=1 to *N* **do**11:    TrajectoryID ← start_trajectory(Configuration*_i_*)12:    Robot*_i_* Operate Done13:    finish_trajectory(TrajectoryID)14:**end for**

#### 4.2.3. Elliptical Filtering

We present an elliptical filtering approach that allows for adjustments to the center, size, and rotation angle to minimize the loss of unnecessary sensor data. The cartographer_ros node was modified for sensor filtering. The z-axis component of the 3D sensor data has a minimal impact on data filtering; therefore, only the x- and y-axis components were considered, and the proposed elliptical filtering was applied to 2D projection. The rotated ellipse used for filtering was mathematically modeled using Equations (1) and (2) for the ellipse and Equation (2) for the rotational transformation matrix. *x* and *y* represent the x- and y-axis, respectively, and *a* and *b* represent the major and minor axis, respectively. $x_{0}$ and $y_{0}$ are the x- and y-coordinate values of the center of the ellipse, respectively. $x_{i}$ and $y_{i}$ represent the *x* and *y* coordinate values within the ellipse defined by Equation (1), while $x_{i}^{\prime }$ and $y_{i}^{\prime }$ represent the x- and y-coordinate values when $x_{i}$ and $y_{i}$ are rotated by θ. The rotational transformation matrix was rotated counterclockwise by θ in a two-dimensional plane. The modeled ellipse equation and sensor data coordinate values were then used to decide whether unnecessary data were included.

(1) $$\frac{{\left(x-x_{0}\right)}^{2}}{a^{2}}+\frac{{\left(y-y_{0}\right)}^{2}}{b^{2}}=1$$

(2) $$0\left(\begin{array}{cc} \cos\theta & -\sin\theta \\ \sin\theta & \cos\theta \end{array}\right)\left(\begin{array}{c} x_{i}\\ y_{i} \end{array}\right)=\left(\begin{array}{c} x_{i}^{\prime }\\ y_{i}^{\prime } \end{array}\right)$$

**Figure 9:**
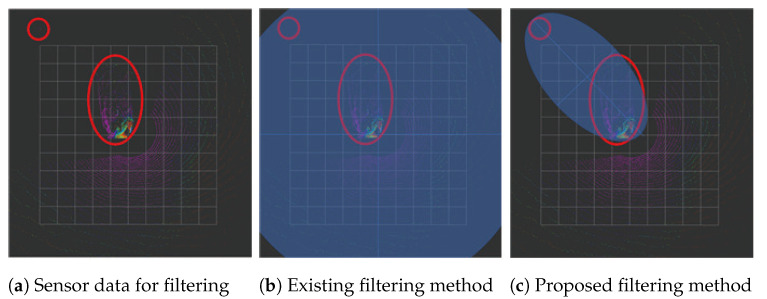
Data filtering comparison. The sensor data indicated by the red circle is subject to filtering.

Figure 9 compares the performance of the existing filtering scheme and the proposed elliptical filtering approach in filtering a specific distribution of the target sensor data. The proposed filtering method resulted in lower sensor data loss. The ‘AddRangeData’ function in the ‘cartographer/mapping/internal/3d/local_trajectory_builder_3d.cc’ file validates, accumulates, and filters the input sensor data, which are established by accessing each sensor’s data independently. Therefore, elliptical filtering is implemented using the ’AddRangeData’ function in the file. The elliptical component can be modified during execution using the existing Lua file without requiring a separate build process.

## 5. Computational Experiments

### 5.1. Sensor Data Preprocessing

**Figure 10:**
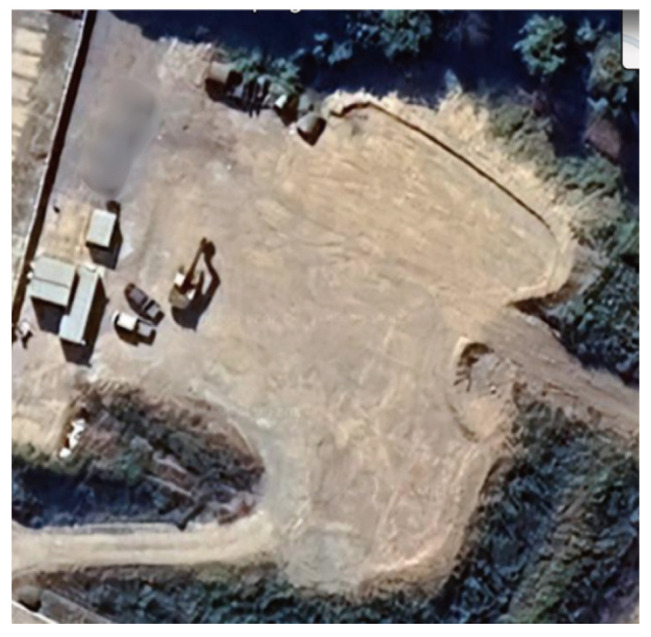
Satellite image.

**Figure 11:**
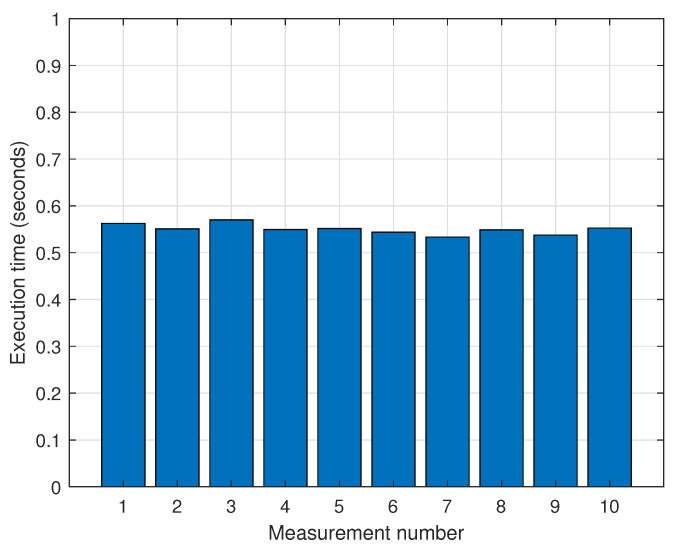
Execution time for sensor data time synchronization.

We validated the sensor data preprocessing by synchronizing the sensor data timestamps and modifying the frame_id field value. We created a bag file, and the site where the bag file was created is shown in the satellite image in Figure 10. Table 2 displays the details of the bag file used for sensor data preprocessing. The bag file had a duration of 379 s and contained PointCloud2 and IMU data. The PointCloud2 and IMU data were published in the /hesai/pandar and /machine_2/imu topics, with timestamps of 1,504,709,606 and 1,712,023,723, respectively. To synchronize the timestamps of the two topics, the timestamp of the IMU data with the higher value was set as the timestamp value of PointCloud2. Table 3 displays the results of the sensor data preprocessing. According to the table, the timestamp value of /machine_2/imu was updated to 1,504,709,606. Furthermore, the frame_id values of the IMU data needed to be updated. Table 2 shows that the frame_id value for the /machine_2/imu topic changed to imu_link. The results demonstrate that the proposed sensor data preprocessing method allows for the synchronization of various sensor data. To evaluate the performance of sensor data preprocessing, we measured the execution time of timestamp synchronization on IMU sensor data. The measurement results in Figure 11 show that it took around 0.55 s on average. This indicates that the operation of the proposed preprocessing is efficient.

### 5.2. Virtual Inertial Data Generation

**Figure 12:**
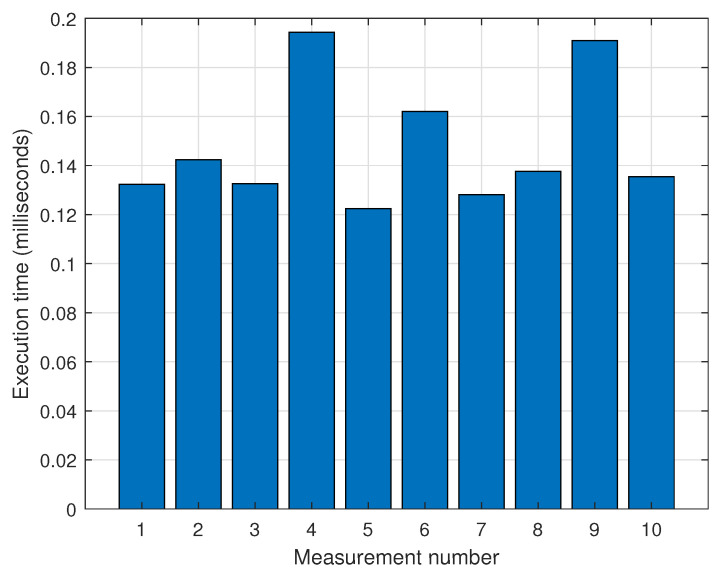
Execution time for virtual inertial data generation.

This section presents the process for validating virtual inertial data generation for 3D SLAM under stationary conditions. The created virtual data must include the x, y, and z values of the linear acceleration field, as well as the timestamp. As it is stationary, both x and y must be set to zero, and z was set to 9.8, which represents the gravitational acceleration. We generated the inertial data, and Table 4 presents the measured data and virtual data generated using the proposed virtual inertial data generation method. The x, y, and z values of the measured linear acceleration results were all zero. However, the table shows that the x and y values were set to 0 and the z values to 9.8. The results confirmed that virtual inertial data, including gravitational acceleration, were generated. We measured the execution time of linear acceleration to evaluate the computation overhead of virtual inertial data generation. The measurement results are presented in Figure 12. The average generating time was approximately 0.15 milliseconds. This result implies that the overhead for virtual inertial data generation is insignificant.

### 5.3. 3D Map Production Offline

**Figure 13:**
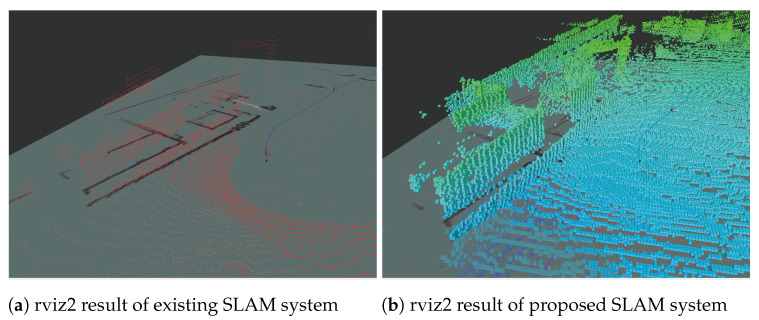
Comparison of rviz2 execution results.

This section presents the validation of the 3D map production function in offline conditions. For generating the 3D map, we used the bag files listed in Table 2. We verified that the 3D terrain map was built using the previously saved bag file in the offline environment and that the generated 3D map was output by the rviz2 node. Figure 13 shows the rviz2 screens of the SLAM system with the proposed 3D map production function and the current SLAM system. The results for the current and proposed SLAM systems are shown in Figure 13a and Figure 13b, respectively. Although the 3D map shown in Figure 13a was poorly created, Figure 13b shows the 3D terrain displayed using rviz2. These results indicate that the proposed 3D map production function can produce 3D maps in an offline environment from a previously created bag file.

### 5.4. Multi-Node SLAM

**Figure 14:**
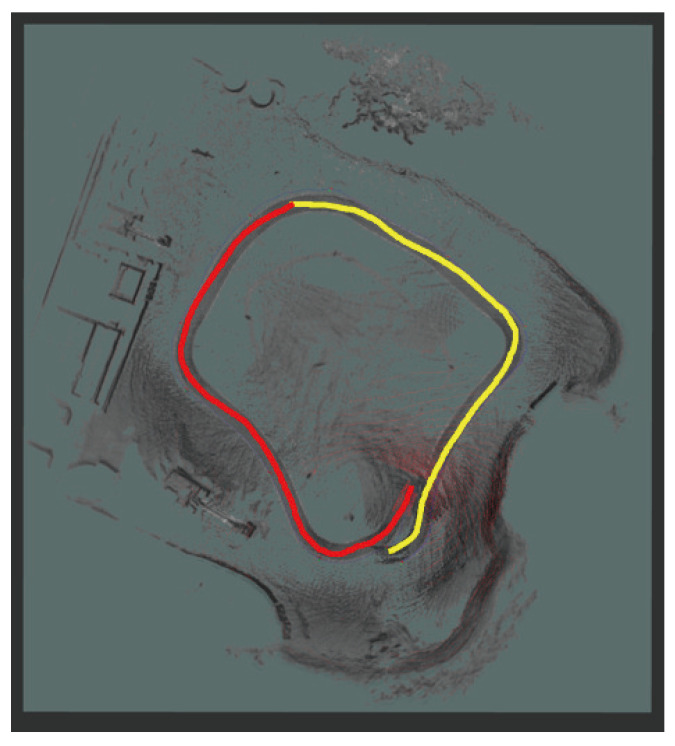
The result of multi-node SLAM execution. The yellow and red lines represent the SLAM execution routes for bag4_split_1 and bag4_split_2, respectively.

To evaluate the feasibility of the multi-node SLAM system’s functionality, we separated the bag files listed in Table 2 into two different files. Table 5 presents information on the split bag files. Multiple trajectories were combined into a single map for multi-node SLAM. The results are shown in Figure 14. The yellow and red lines in the figure represent the SLAM execution routes for bag4_split_1 and bag4_split_2, respectively. We compared the results with those shown in Figure 10, which depicts a satellite image of the site where the bag files were recorded. By comparing the outline of the location and the location of the buildings, we confirmed that the results of the multi-node SLAM system were similar to the actual location. Without multi-node SLAM functionality, only half of the map in Figure 14 could be generated from a separate bag file. The bag file for the route on the red line would have generated the left side of the map, whereas the bag file for the route on the yellow line would have generated the right side.

### 5.5. Elliptical Filtering

**Figure 15:**
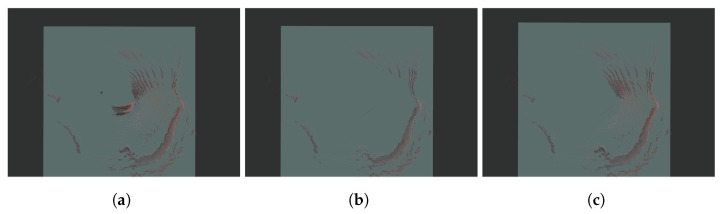
rviz2 execution results with different filtering schemes. (**a**) Without filtering. (**b**) With distance-based filtering. (**c**) With the proposed filtering method.

The performance of the proposed elliptical filtering scheme was evaluated. For the bag file captured on-site in Figure 10, we used no filtering, distance-based filtering, or the suggested elliptical filtering. We used the /scan_matched_points2 topic represented by a red point in rviz2 to evaluate the effectiveness of the different filtering schemes visually. Cartographer filters the input data and publishes the results using the /scan_matched_points2 topic, which is a message in the format of PointCloud2. The results of the various filtering schemes are displayed in Figure 15. Because no filtering was used, all the measured sensor data are shown in Figure 15a. The results of using distance-based filtering in SLAM with the distance set to 10 are shown in Figure 15b. In the figure, the sensor data included in a circle with a radius of 10 centered on the origin were filtered. The results obtained using the proposed filtering method are shown in Figure 15c. The origin of the ellipse was at (5, 0). and it rotated 60° clockwise with major and minor axes of 7 and 5, respectively. In contrast to the results of the distance-based filtering, the sensor data measured from the right-hand side are displayed as the results of the proposed filtering method. Consequently, the proposed filtering method maintains essential sensor data while eliminating extraneous data.

**Figure 16:**
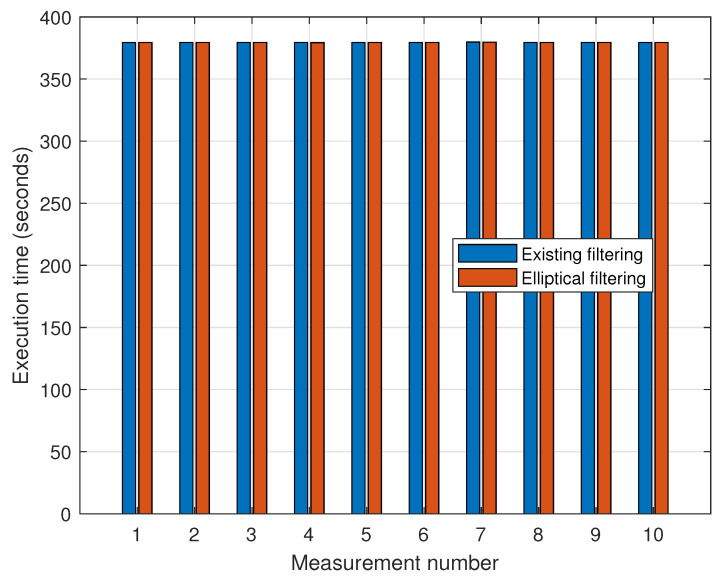
Comparison of execution time between existing filtering and elliptical filtering methods.

Figure 16 depicts the execution times for elliptical filtering and the existing distance-based filtering method. The average execution times for the existing and proposed filtering methods were 379.46 s and 379.42 s, respectively. As a result, the two filtering approaches have about the same computing overhead.

## 6. Conclusions

This study implemented a SLAM system that improves aspects of the current Cartographer version’s limitations by incorporating five additional features. Various sensor-related issues can be addressed through the implemented virtual inertial data generator and sensor data time synchronization scheme. This functionality was enhanced by integrating Cartographer with OctoMap to perform real-time 3D mapping. In addition, the feasibility of multi-node SLAM for increasing scalability was investigated. Elliptical filtering was proposed for filtering unnecessary sensor data. The proposed approaches provide significant benefits for improving autonomous robot movement. Multi-node SLAM, in particular, improves robotic systems’ scalability, while real-time 3D terrain mapping allows for more precise environmental awareness. Sensor data time synchronization and virtual inertial data generation have average execution times of 0.55 s and 0.15 milliseconds, respectively. This implies that the two schemes have a modest computational overhead. These technologies can make a substantial contribution to increasing the efficiency of industrial robots.

## Figures and Tables

**Table 1:** Comparisons with SLAM frameworks.

	SLAM Classification		Mapping		Multi-Node
	Visual SLAM	LiDAR SLAM	2D	3D	
ORB-SLAM	🗸			🗸	
Hector SLAM		🗸	🗸		
RTAB-Map	🗸		🗸	🗸	
Cartographer		🗸	🗸	🗸	
Proposed scheme		🗸	🗸	🗸	🗸

**Table 2:** Bag file information.

Topic	Type	Count	Header	
			stamp.sec	frame_id
/hesai/pandar	sensor_msgs/msg/Pointcloud2	3794	1,504,709,606	PandarQT
/machine_2/imu	sensor_msgs/msg/Imu	3798	1,712,023,723	

**Table 3:** Sensor data preprocessing result.

	header.stamp.sec	header.frame_id
Unpreprocessed	1,712,023,723	“”
Preprocessed	1,504,709,606	“imu_link”

**Table 4:** The measured and virtually generated linear acceleration results.

Measured			Virtual		
x	y	z	x	y	z
0	0	0	0	0	9.8

**Table 5:** Split bag file information.

File	Topic	Type	Count
bag4_split_1	/imu	sensor_msgs/msg/Imu	2109
	/hesai/pandar	sensor_msgs/msg/PointCloud2	2115
bag4_split_2	/imu	sensor_msgs/msg/Imu	1662
	/hesai/pandar	sensor_msgs/msg/PointCloud2	1664

## Data Availability

The data are contained within the article.

## Abbreviations

The following abbreviations are used in this manuscript:

SLAM	Simultaneous localization and mapping
ROS	Robot Operating System
LiDAR	Light detection and ranging
IMU	Inertial measurement unit
TF	Transform
